# *Helicobacter pylori*-induced IL-33 modulates mast cell responses, benefits bacterial growth, and contributes to gastritis

**DOI:** 10.1038/s41419-018-0493-1

**Published:** 2018-04-25

**Authors:** Yi-pin Lv, Yong-sheng Teng, Fang-yuan Mao, Liu-sheng Peng, Jin-yu Zhang, Ping Cheng, Yu-gang Liu, Hui Kong, Ting-ting Wang, Xiao-long Wu, Chuan-jie Hao, Weisan Chen, Shi-ming Yang, Yong-liang Zhao, Bin Han, Qiang Ma, Quan-ming Zou, Yuan Zhuang

**Affiliations:** 1 National Engineering Research Centre of Immunological Products, Department of Microbiology and Biochemical Pharmacy, College of Pharmacy, Third Military Medical University, Chongqing, China; 2 La Trobe Institute of Molecular Science, School of Molecular Science, La Trobe University, Bundoora, Victoria 3085 Australia; 3 Department of Gastroenterology, XinQiao Hospital, Third Military Medical University, Chongqing, China; 4 Department of General Surgery and Centre of Minimal Invasive Gastrointestinal Surgery, Southwest Hospital, Third Military Medical University, Chongqing, China; 5 Department of Pharmacy, Affiliated Hospital of North Sichuan Medical College, Nanchong, Sichuan Province China

## Abstract

Interleukin (IL)-induced inflammatory responses are critical for the pathogenesis of *Helicobacter pylori* (*H. pylori*)-induced gastritis. IL-33 represents a recently discovered proinflammatory cytokine involved in inflammatory diseases, but its relevance to *H. pylori*-induced gastritis is unknown. Here, we found that gastric IL-33 mRNA and protein expression were elevated in gastric mucosa of both patients and mice infected with *H. pylori*, which is positively correlated with bacterial load and the degree of gastritis. IL-33 production was promoted via extracellular regulated protein kinases (ERK) signaling pathway activation by gastric epithelial cells in a *cagA*-dependent manner during *H*. *pylori* infection, and resulted in increased inflammation and bacteria burden within the gastric mucosa. Gastric epithelial cell-derived IL-33 promoted TNF-α production from mast cells in vitro, and IL-33 increased TNF-α production in vivo. Increased TNF-α inhibited gastric epithelial cell proliferation, conducing to the progress of *H*. *pylori*-associated gastritis and bacteria colonization. This study defined a patent regulatory networks involving *H. pylori*, gastric epithelial cell, IL-33, mast cell, and TNF-α, which jointly play a pathological effect within the gastric circumstances. It may be a valuable strategy to restrain this IL-33-dependent pathway in the treatment of *H. pylori*-associated gastritis.

## Introduction

*Helicobacter pylori (H. pylori)* is a Gram-negative bacteria, which has infected more than half of the world’s population. Colonization and long-term infection of *H. pylori* in the human stomach almost always leads to chronic gastritis, even peptic ulceration or gastric tumor^1^. Inflammatory reaction to *H. pylori* infection shows special characteristics rarely seen in other organs or biological systems. In addition, a mixed acute and chronic inflammatory reaction takes place simultaneously during *H. pylori* infection, where a variety of immune cells infiltrate the mucosa in a characteristic manner^2–4^.

Although mast cells have been known for their notable role in anaphylaxis, they play a part in innate immune reactions against bacterial infection by secreting cellular factors^5^. Infiltration of mast cells is limited to a certain extent in normal mucosa. It is often elevated during inflammation^6^. Recently, a few studies have found that mast cells also participated in chronic gastritis and they increased in number as the disease worsened^7^.

Interleukin-33 (IL-33) pertains to the IL-1 cytokine family and participates in regulating the innate and adaptive immune responses^8^, especially during some allergic, autoimmune, and inflammatory diseases^9,10^. Some studies have revealed that IL-33 is a tissue-derived nuclear cytokine mainly produced by endothelial cells, epithelial cells, fibroblast-like cells, and myofibroblasts in human and mouse^11^. Interestingly, it has been reported that gastric epithelial cells can secrete IL-33^12^, and IL-33 can promote mast cells to release serine proteases (chymase and tryptase)^13^, as well as proinflammatory mediators to augment the effects of IgE^14^.

Here we report that *H. pylori* infection can induce gastric epithelium damage and necrosis, which triggered IL-33 release from primary gastric epithelial cells. And then, IL-33 enhances mast cell-derived tumor necrosis factor-alpha (TNF-α) secretion in gastritis. In turn, TNF-α aggravates the inflammation and *H. pylori* colonization; furthermore, IL-33 inhibits gastric epithelial cell renewal and promotes gastritis progress. These findings provide further insight into understanding and potentially treatment of *H. pylori*-associated gastritis.

## Results

### IL-33 is increased in gastric mucosa of *H. pylori*-infected patients and mice

**Fig. 1:**
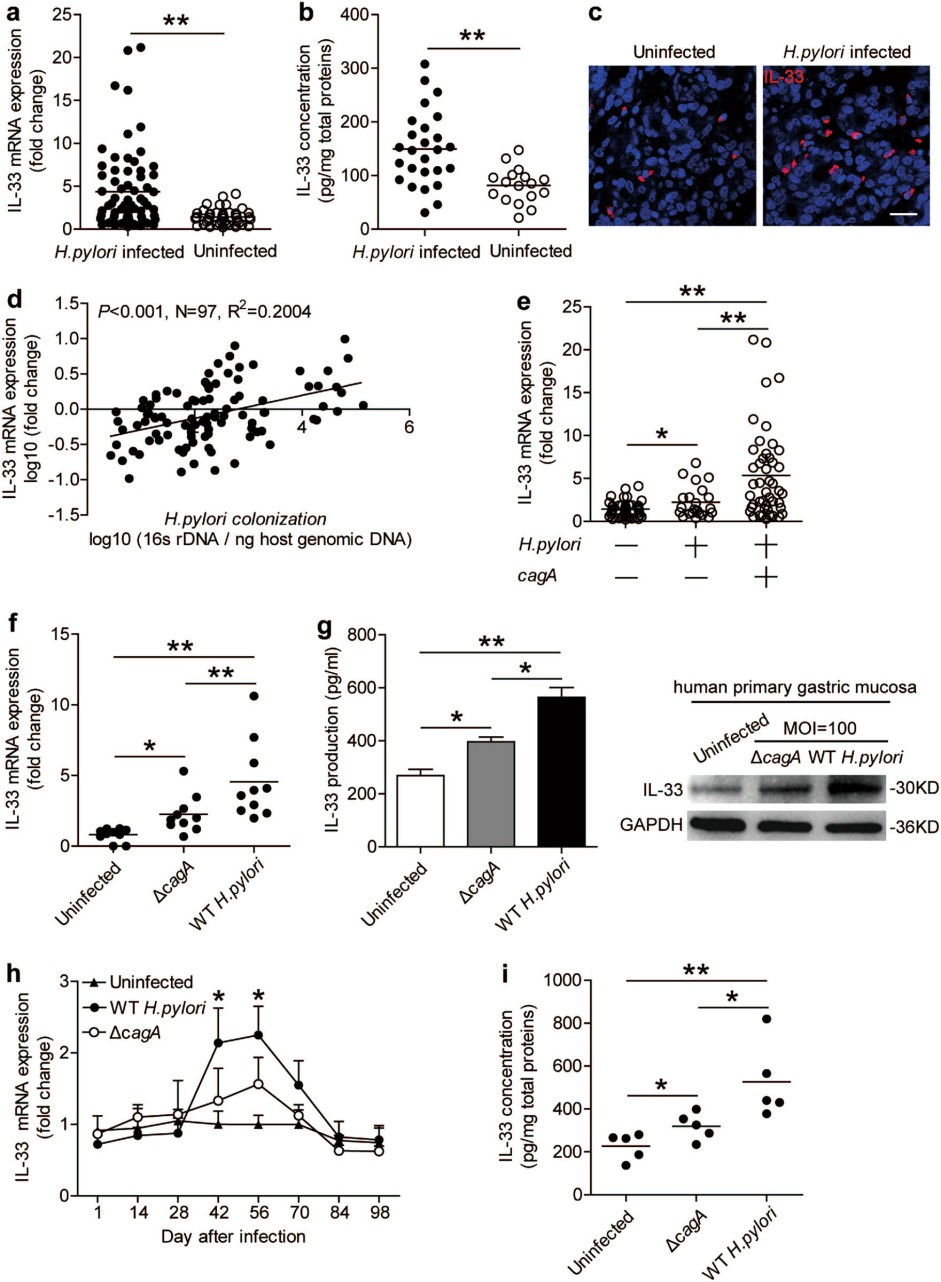
Increased IL-33 is detected in gastric mucosa of *H. pylori*-infected patients and mice. **a,** **b** The comparison of IL-33 mRNA **a** and protein concentration **b** in gastric mucosa of *H. pylori*-infected (*n* = 63) and uninfected donors (*n* = 48) was shown. **c** Representative immunofluorescence images showing IL-33^+^ cells infiltrating in gastric mucosa of *H. pylori*-infected and uninfected donors. Red, IL-33; and blue, DAPI-stained nuclei. Scale bars: 20 μm. **d** The correlation of IL-33 level and *H. pylori* colonization was analyzed. **e** The expression of IL-33 mRNA in gastric mucosa of uninfected (*n* = 48), *cagA*^−^ *H. pylori*-infected (*n* = 22), and *cagA*^+^ *H. pylori*-infected (*n* = 41) donors was measured. **f**, **g** Expression of IL-33 mRNA (**f**) or IL-33 protein (**g**) in uninfected, *ΔcagA-*strain-infected, and WT *H. pylori-*strain-infected gastric mucosa from uninfected donors were measured (*n* = 10) or analyzed by ELISA (*n* = 3) and western blot. **h** Dynamic changes of IL-33 mRNA expression in WT *H. pylori-*strain-infected, *ΔcagA-*strain-infected, and uninfected C57BL/6 wild-type (WT) mice. **i** Concentrations of IL-33 protein in gastric mucosa of WT *H. pylori-*strain-infected, *ΔcagA-*strain-infected, and uninfected mice on day 56 p.i. were compared. The mean values are represented with horizontal bars in **a**, **b**, **e**, **f** and **i**. Each patient or mouse is represented with a dot or ring in **a**, **b**, **d**, **e**, **f** and **i**. Each group has five mice at per time point in **h**. *P < 0.05 and **P < 0.01

First, we analyzed the IL-33 levels in *H. pylori*-infected and -uninfected gastric mucosa to evaluate the potential role of IL-33 in *H. pylori*-associated immunopathogenesis, and found that, compared with uninfected donors, the levels of IL-33 mRNA (Fig. 1a) and protein (Fig. 1b) in gastric mucosa of *H. pylori*-infected patients were significantly higher, a result that was also supported by immunofluorescence staining (Fig. 1c). Next, we also found a positive correlation between IL-33 and *H. pylori* colonization (Fig. 1d), suggesting that *H. pylori* infection could induce the increase of IL-33.

It has previously been shown that, *cagA*, a key virulence factor of *H. pylori*, is closely related with the development of *H. pylori*-associated gastritis^15^. Interestingly, compared with *cagA*-negative individuals, IL-33 expression was significantly higher in *cagA*-positive patients (Fig. 1e). We next examined the expression of IL-33 in human primary gastric mucosa stimulated with *H. pylori*. The levels of IL-33 mRNA (Fig. 1f) and protein (Fig. 1g) were significantly upregulated with WT *H. pylori-*strain infection compared with either no infection or infection with a *ΔcagA-*strain. The soluble form of the IL-33 cell surface receptor, sST2, acts as endogenous inhibitor of the extracellular functions of IL-33^16^. Hence, it is necessary to detect the production of sST2 in local environment. However, we found no change of sST2 expression in gastric mucosa between *H. pylori*-infected patients and uninfected donors (Supplementary figure 1d). So, the IL-33 bioactivity would not be limited by sST2 due to *H. pylori-*infected or not.

In addition, we detected IL-33 expression in gastric mucosa of mice infected with *H. pylori*. Consistent with our findings in humans, IL-33 mRNA (Fig. 1h) and protein (Fig. 1i) increase was only detected in gastric mucosa of WT *H. pylori-*strain-infected mice, reaching a peak 56 days post infection (p.i.), indicating a crucial role for *cagA* in the induction of IL-33 during *H. pylori* infection.

### *H. pylori* infection induces gastric epithelial cells to produce IL-33 in a *cagA*-dependent manner

**Fig. 2:**
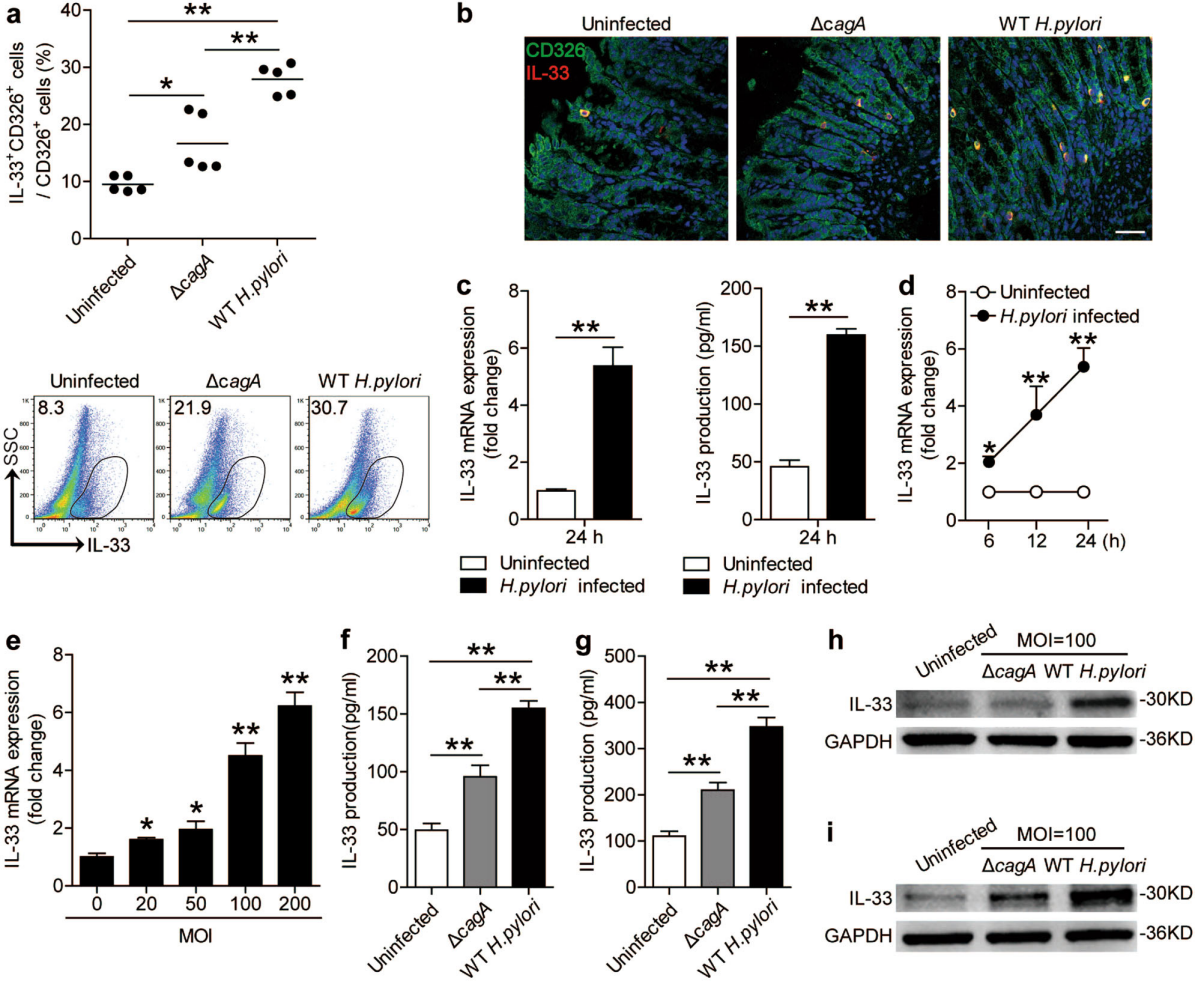
*H. pylori*-infected gastric epithelial cells produce IL-33. **a** The percentage of IL-33^+^ cells within total CD326^+^ gastric epithelial cells in gastric mucosa of WT *H. pylori-*strain-infected, *ΔcagA-*strain-infected, and uninfected mice on day 56 p.i. were compared. **b** Representative immunofluorescence staining images showed IL-33^+^CD326^+^ cells in gastric mucosa of WT *H. pylori-*strain-infected, *ΔcagA-*strain-infected, and uninfected mice on day 56 p.i. Green, CD326; red, IL-33; and blue, DAPI-stained nuclei. Scale bars: 50 μm. **c** IL-33 mRNA expression in *H. pylori*-infected and uninfected AGS cells were compared (*n* = 3). **d**, **e** IL-33 mRNA expression in *H. pylori*-infected and uninfected AGS cells at different time point (MOI = 100) **d** or with different MOI (24 h) **e** were compared (*n* = 3). **f**, **h** IL-33 protein in WT *H. pylori-*strain-infected, *ΔcagA-*strain-infected, and uninfected AGS cells (MOI = 100, 24 h) were analyzed by ELISA (*n* = 3) **f** and western blot **h**. **g**, **i** IL-33 protein in uninfected, *ΔcagA-*strain-infected, and WT *H. pylori-*strain-infected primary gastric epithelial cells from uninfected donors (MOI = 100, 24 h) were analyzed by ELISA (*n* = 3) **g** and western blot **i**. The horizontal bars in **a** represent mean values. Each dot in **a** represents one mouse. *P < 0.05 and **P < 0.01

It has previously been reported that IL-33 can also be produced by endothelial, epithelial, and fibroblast-like cells^17^. Using our mouse model with *H. pylori* infection, we found IL-33 expression in CD326^+^ gastric epithelial cells by flow cytometry (Fig. 2a) and immunofluorescence staining (Fig. 2b), and this was most noticeable when infected with WT *H. pylori-*strain. These data suggest that, in *H. pylori*-infected gastric mucosa, IL-33 could be produced from gastric epithelial cells.

Next, to further determine whether IL-33 is induced from *H. pylori*-infected gastric epithelial cells, we stimulated AGS cells, an immortalized human gastric epithelial cell line, with *H. pylori*, and found that *H. pylori-*infected AGS cells were able to potently increase the levels of IL-33 mRNA and protein (Fig. 2c) in a time-dependent (Fig. 2d), as well as a dose-dependent (Fig. 2e) manner, and this IL-33 induction was most noticeable when using a WT *H. pylori*-strain compared with *ΔcagA-*strain (Figs. 2f, h). Notably, compared with no infection or infection with *ΔcagA-*strain, WT *H. pylori-*strain-infected human primary gastric epithelial cells were also able to potently increase IL-33 production (Figs. 2g, i). These data altogether suggest that, in *H. pylori*-infected gastric mucosa, *H. pylori* infection induces gastric epithelial cells to produce IL-33.

### *H. pylori* stimulates gastric epithelial cells to induce IL-33 production via extracellular regulated protein kinases (ERK) pathway

**Fig. 3:**
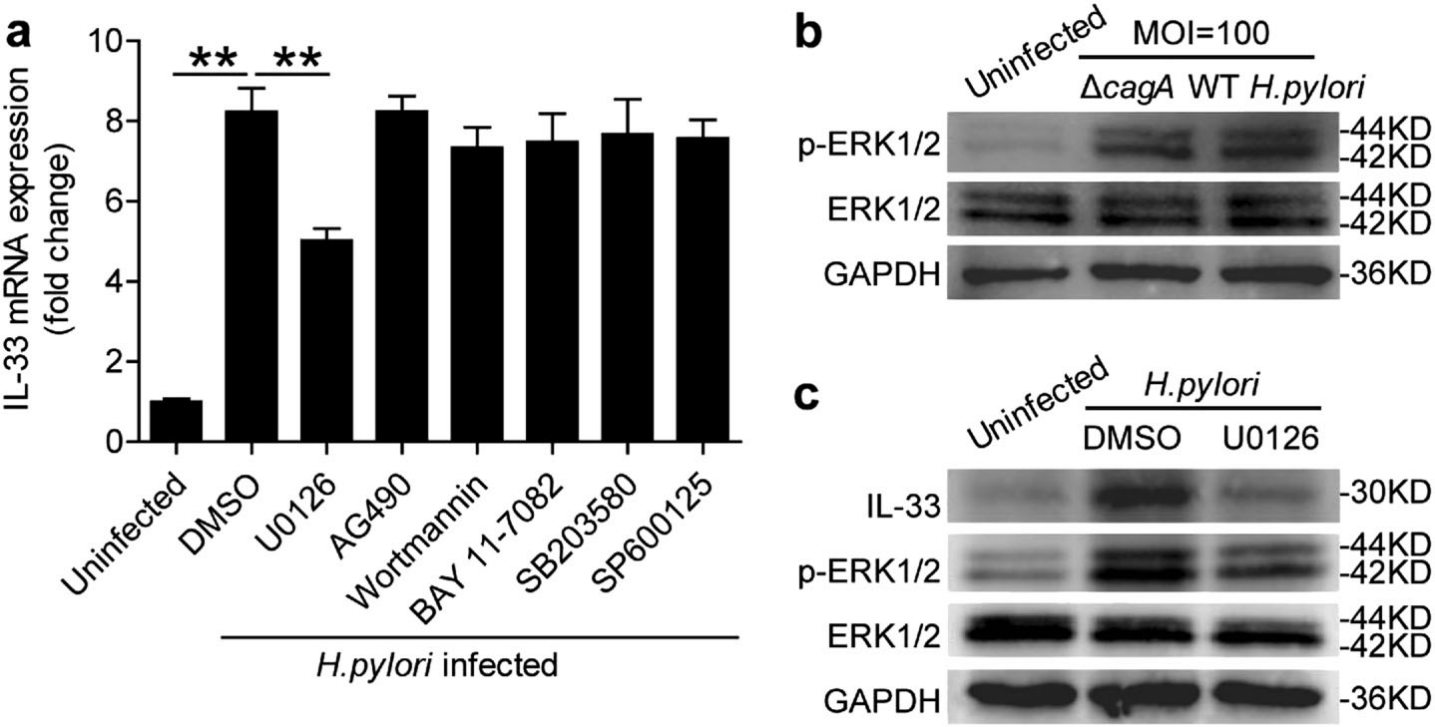
*H. pylori* induces IL-33 production of gastric epithelial cells via ERK pathway. **a** AGS cells were pre-treated with U0126 (an ERK inhibitor), AG490 (a JAK inhibitor), Wortmannin (a PI3K inhibitor), BAY 11-7082 (an IκBα inhibitor), SB203580 (a MAPK inhibitor), or SP600125 (a JNK inhibitor) and then stimulated with WT *H. pylori-*strain (MOI = 100) for 24 h. IL-33 mRNA expression in AGS cells was compared (*n* = 3). **b,** **c** AGS cells were stimulated with WT *H. pylori-* or *ΔcagA-*strain (MOI = 100) for 6 h **b**, or were pre-treated with U0126 (an ERK inhibitor) and then stimulated with WT *H. pylori-*strain (MOI = 100) for 6 h **c**. ERK1/2 and p-ERK1/2 proteins were analyzed by western blots. **P < 0.01

To see which signaling pathways might operate in the induction of IL-33 from gastric epithelial cells, first we used corresponding inhibitors to treat AGS cells, and then stimulated AGS cells with *H. pylori*. We found that only blocking ERK pathway with U0126, ERK pathway-associated inhibitor, effectively suppressed IL-33 expression in *H. pylori*-infected gastric epithelial cells (Fig. 3a). Furthermore, ERK1/2, a direct ERK pathway downstream substrate, was predominantly phosphorylated in gastric epithelial cells after stimulated with *H. pylori*, and this was more noticeable when infected with a WT *H. pylori-*strain compared with *ΔcagA-*strain (Fig. 3b), and this phosphorylation was abolished when ERK signal transduction pathway was blocked with inhibitor U0126 (Fig. 3c), implying that activation of ERK signaling pathway is crucial for IL-33 induction by *H. pylori*-infected gastric epithelial cells.

### IL-33 increases TNF-α production, inflammation, and bacterial burden in gastric mucosa during *H. pylori* infection

**Fig. 4:**
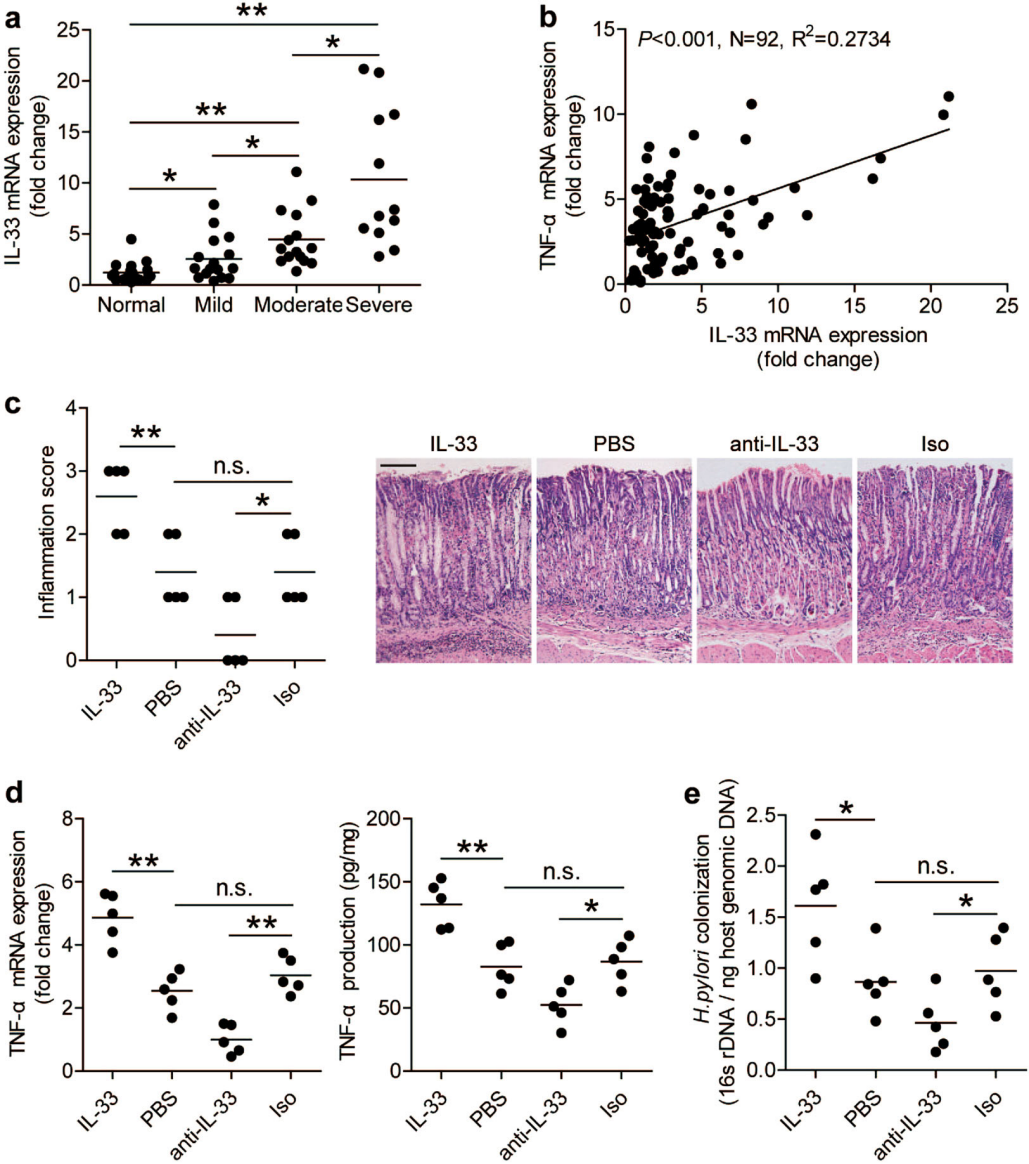
IL-33 increases TNF-α production, inflammation, and bacterial burden in gastric mucosa during *H. pylori* infection. **a** IL-33 mRNA expression in gastric mucosa of *H. pylori*-infected patients with normal gastric histopathology (*n* = 20), or with mild (*n* = 16), moderate (*n* = 15), severe inflammation (*n* = 12) was compared. **b** The correlation between IL-33 expression and TNF-α expression was analyzed. **c** Histological scores of inflammation in gastric antra of WT *H. pylori-*strain-infected mice injected with PBS control or IL-33, or IL-33 neutralizing antibody or corresponding isotype control antibody on day 56 p.i. were compared. H&E staining, scale bars: 100 μm. **d** Expression of TNF-α mRNA or production of TNF-α protein in gastric mucosa of WT *H. pylori-*strain-infected mice injected with PBS control or IL-33, or IL-33 neutralizing antibody or corresponding isotype control antibody on day 56 p.i. were compared or analyzed by ELISA. **e** The bacterial colonization in gastric mucosa of WT *H. pylori-*strain-infected mice injected with PBS control or IL-33, or IL-33 neutralizing antibody or corresponding isotype control antibody on day 56 p.i. were compared. The horizontal bars in **a**, **c**, **d**, and **e** represent mean values. Each dot represents one subject or mouse. *P < 0.05, **P < 0.01, and n.s., *P* > 0.05

To evaluate the possible biological effects of IL-33 in *H. pylori*-associated immunopathogenesis in vivo, we compared the levels of IL-33 expression in gastric mucosa with different severity of gastritis, and found that the expression of IL-33 was positively correlated with the severity of gastritis (Fig. 4a), and a positive correlation between IL-33 and TNF-α was observed (Fig. 4b). This led us to hypothesize that, during *H*. *pylori* infection, IL-33 might exert proinflammatory effects and promote TNF-α production and, as a result, lead to gastritis.

To test this hypothesis in vivo, a series of loss- and gain-of-function experiments involving IL-33 were performed, the inflammatory response was evaluated in gastric mucosa on day 56 p.i. Neutralization of IL-33 significantly reduced gastric inflammation (Fig. 4c) and TNF-α production (Fig. 4d). Conversely, injection of IL-33 significantly increased gastric inflammation (Fig. 4c) and TNF-α production (Fig. 4d). Finally, consistent with IL-33 contributing to pathology, IL-33 appears to benefit bacterial growth as provision of exogenous IL-33 increased bacterial burden, whereas IL-33 blockade reduced *H. pylori* colonization (Fig. 4e). Collectively, these results suggest that IL-33 promoted TNF-α production, inflammation, and bacterial colonization during *H. pylori* infection in vivo.

### Gastric epithelial cell-derived IL-33 promotes TNF-α production from mast cells during *H. pylori* infection

**Fig. 5:**
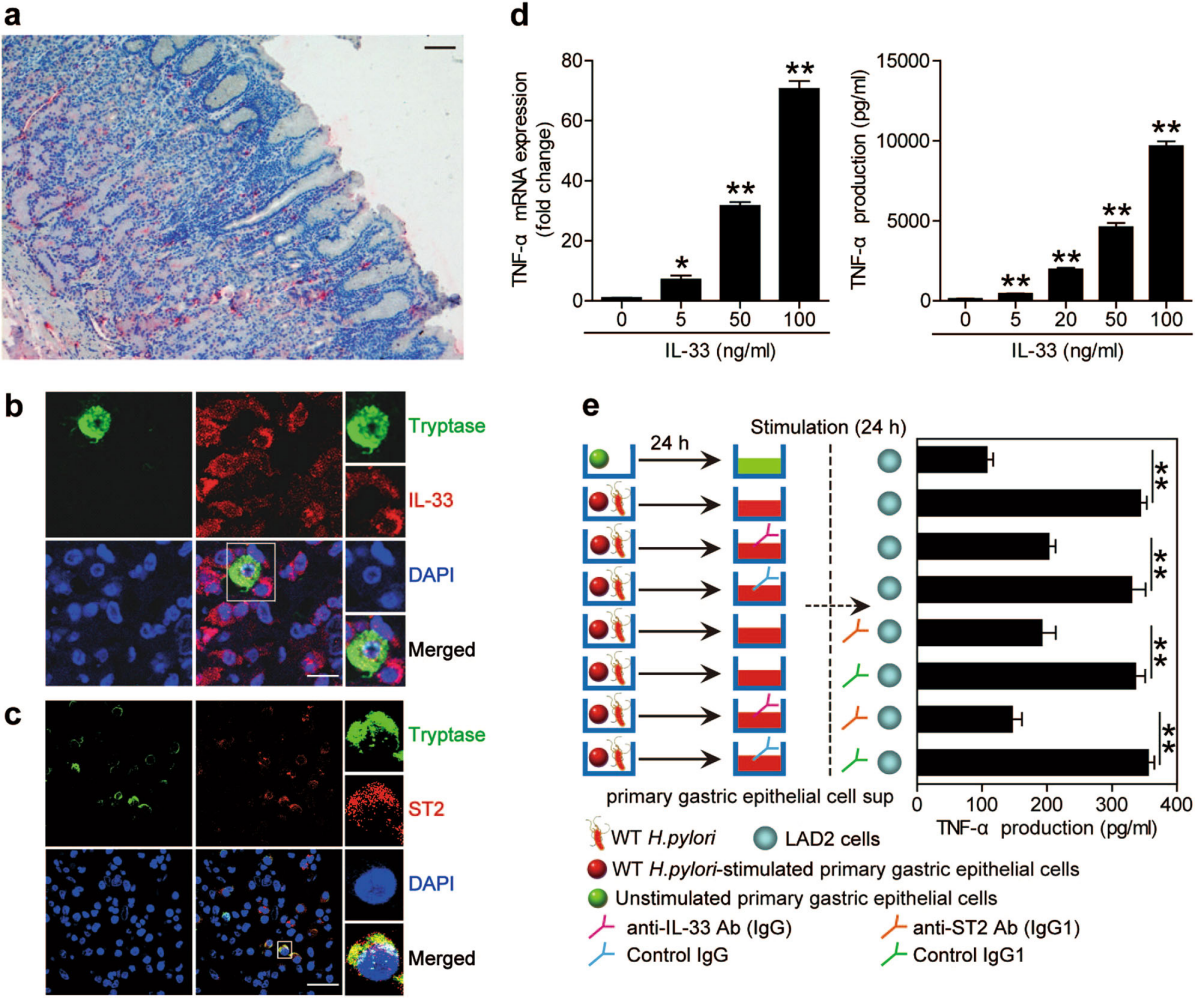
Gastric epithelial cell-derived IL-33 promotes TNF-α production from mast cells during *H. pylori* infection. **a** Representative immunohistochemistry images showing tryptase^+^ (red) mast cell infiltration in gastric mucosa of *H. pylori*-infected patients. Scale bars: 50 μm. **b** Representative immunofluorescence images showing tryptase^+^ mast cell and IL-33^+^ cell interactions in gastric mucosa of *H. pylori*-infected patients. Green, tryptase; red, IL-33; and blue, DAPI-stained nuclei. Scale bars: 10 μm. **c** Representative immunofluorescence images showing tryptase^+^ST2^+^ mast cell infiltration in gastric mucosa of *H. pylori*-infected patients. Green, tryptase; red, ST2; and blue, DAPI-stained nuclei. Scale bars: 50 μm. **d** LAD2 cells were stimulated with IL-33. Expression of TNF-α mRNA or TNF-α protein in LAD2 cells were compared (*n* = 3) or analyzed by ELISA (*n* = 3). **e** TNF-α production from LAD2 cells stimulated with different culture supernatants from primary gastric epithelial cells was measured by ELISA as represented in Methods performed (*n* = 3). *P < 0.05 and **P < 0.01

IL-33 is known to induce the production of various proinflammatory cytokines from mast cells during inflammation^18^. We were therefore interested to know if IL-33 modulated mast cell responses in gastric mucosa during *H. pylori* infection. To begin, we found that a mast cell infiltration (Fig. 5a) and the colocalization of mast cells and IL-33^+^ cells (Fig. 5b) in *H. pylori*-infected gastric mucosa. Moreover, the expression of IL-33 receptor, ST2, was confirmed to merge with tryptase staining on mast cells in *H. pylori*-infected gastric mucosa (Fig. 5c), suggesting that mast cells are a major target of IL-33 action within the inflamed gastric mucosa during *H. pylori* infection. Certainly, some other immune cells could express ST2 in gastritis, including CD8^+^ lymphocytes (Supplementary figure 2c).

To show the regulation of TNF-α by IL-33 during *H. pylori* infection in vitro (Fig. 5d), we stimulated mast cells with IL-33 to observe change of TNF-α. Interestingly, IL-33 significantly induced mast cell line LAD2 (Fig. 5d) and BMMCs (supplementary Figure 1) to produce TNF-α in a dose-dependent manner. It is well known that CD8^+^ lymphocytes are also as a source of TNF-α, so we detected TNF*-*α production from the CD8^+^ lymphocytes after stimulation by IL-33. Though IL-33 could induce CD8^+^ lymphocytes to produce TNF*-*α, it was less than that from mast cells (supplementary figure 2d). In our study, mast cells might be the main source of TNF*-*α in gastric mucosa.

Moreover, to evaluate the contribution of IL-33, derived from *H. pylori*-infected-gastric epithelial cells, to mast cell TNF-α production, mast cell line LAD2 was incubated in the culture supernatants collected from primary gastric epithelial cells stimulated with WT *H. pylori-*strain. The results showed that such supernatants induced significantly more TNF-α production by LAD2 mast cells than those from unstimulated gastric epithelial cells (Fig. 5e), and this effect was lost upon pre-treatment with IL-33 neutralizing Abs or ST2 blocking Abs (Fig. 5e). Collectively, these results therefore suggest that a gastric epithelial cell–IL-33–mast cell axis contributes to TNF-α production within the inflamed gastric mucosa during *H. pylori* infection.

### TNF-α promotes inflammation and bacterial colonization in gastric mucosa during *H. pylori* infection

**Fig. 6:**
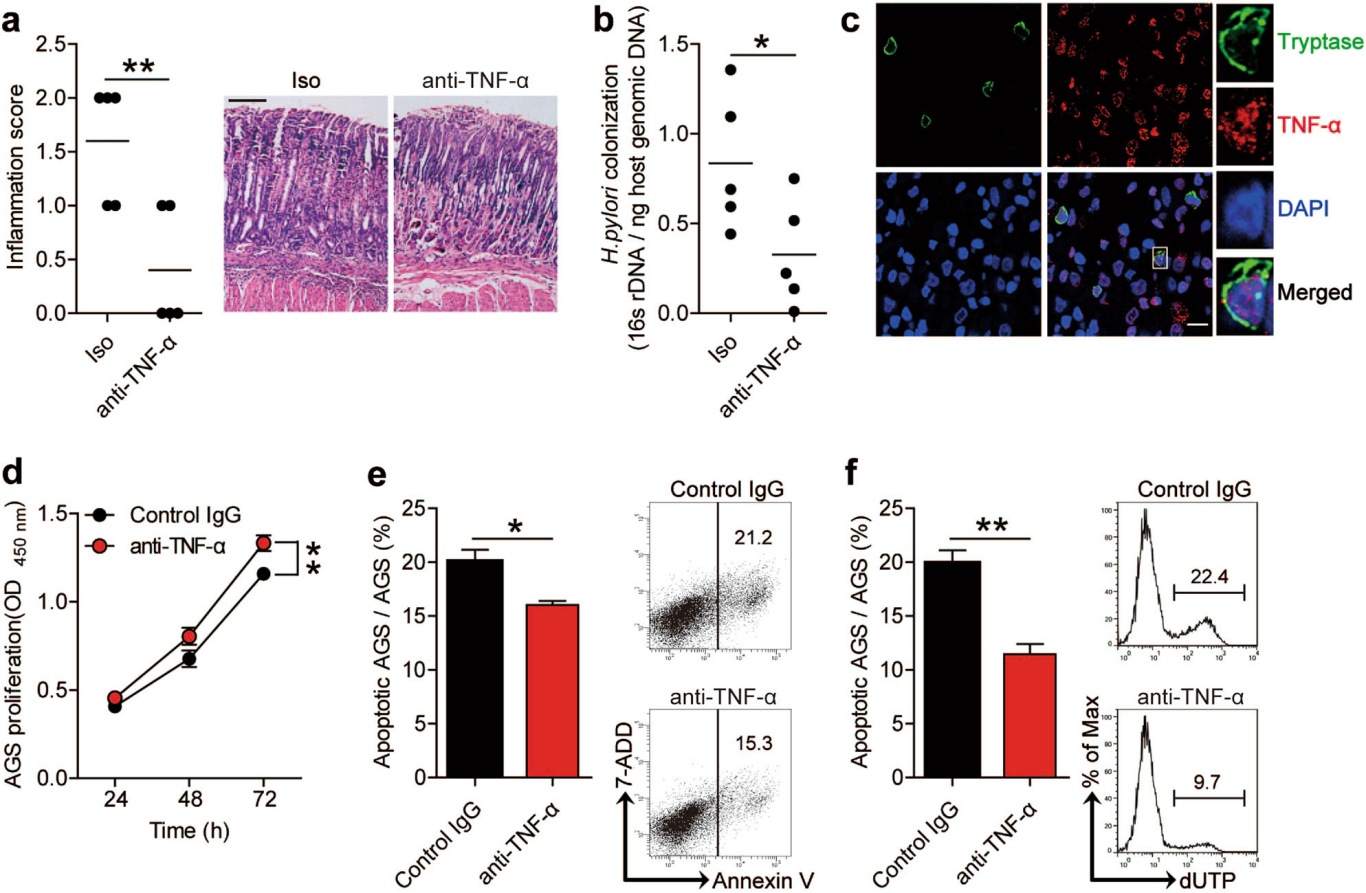
TNF-α promotes inflammation and bacteria colonization in gastric mucosa during *H. pylori* infection. **a** Histological scores of inflammation in gastric antra of WT *H. pylori-*strain-infected mice injected with Abs against TNF-α or corresponding isotype control Ab on day 56 p.i. were compared. H&E staining, scale bars: 100 μm. **b** The bacteria colonization in gastric mucosa of WT *H. pylori-*strain-infected mice injected with Abs against TNF-α or corresponding isotype control Ab on day 56 p.i. were compared. **c** Representative immunofluorescence staining images showed tryptase^+^TNF-α^+^ infiltration in gastric mucosa of *H. pylori*-infected patients. Green, tryptase; red, TNF-α; and blue, DAPI-stained nuclei. Scale bars: 10 μm. **d-f** AGS cells were stimulated with LAD2 conditional supernatants plus control IgG1 or TNF-α neutralizing Abs, as described in Materials and methods section. The proliferation **d** of AGS cells were analyzed by CCK-8 detection (*n* = 3). The apoptosis of AGS cells were analyzed by Annexin V **e** and deoxyuridine triphosphate nucleotides (dUTP) **f** detection (*n* = 3). The mean values are represented with horizontal bars in **a** and **b**. Each mouse is represented with a dot or ring in **a** and **b**. *P < 0.05 and **P < 0.01

Furthermore, in order to approach the potential biological effects of mast cell-derived TNF-α in *H. pylori*-associated immunopathogenesis in vivo, we blocked TNF-α and evaluated the inflammatory response and *H. pylori* colonization in gastric mucosa on day 56 p.i. (when TNF-α mRNA increase in gastric mucosa in WT *H. pylori-*strain-infected mice reaches its peak; supplementary Figure 3d). We found that neutralization of TNF-α significantly reduced gastric inflammation (Fig. 6a) and *H. pylori* colonization (Fig. 6b), suggesting that TNF-α (most likely derived from tryptase^+^ mast cells; Fig. 6c) has effects of promoting inflammation and bacteria colonization during *H. pylori* infection in vivo.

Gastric epithelial cells are known to be critically important in both host defense and mucosal barrier maintenance. We hypothesized that TNF-α might exert pathological effects during *H. pylori* infection by disturbing function of gastric epithelial cells. Then we stimulated AGS cells with TNF-α and found that TNF-α efficiently inhibited gastric epithelial cell proliferation (supplementary Figure 2a) and promoted gastric epithelial cell apoptosis assessed by Annexin V (supplementary Figure 2b) and deoxyuridine triphosphate nucleotides (dUTP) (supplementary Figure 2c) detection. To further test whether mast cell-derived TNF-α play a role in this process, we stimulated AGS cells with the second mast cell culture supernatants with or without neutralizing Abs against TNF-α. Interestingly, blocking TNF-α efficiently promoted gastric epithelial cell proliferation (Fig. 6d) and inhibited gastric epithelial cell apoptosis (Fig. 6f). Collectively, our data indicate that, during *H. pylori* infection, mast cell-derived TNF-α might play an essential role in promoting bacteria colonization and inflammation by inhibiting gastric epithelial cell renewal.

## Discussion

**Fig. 7:**
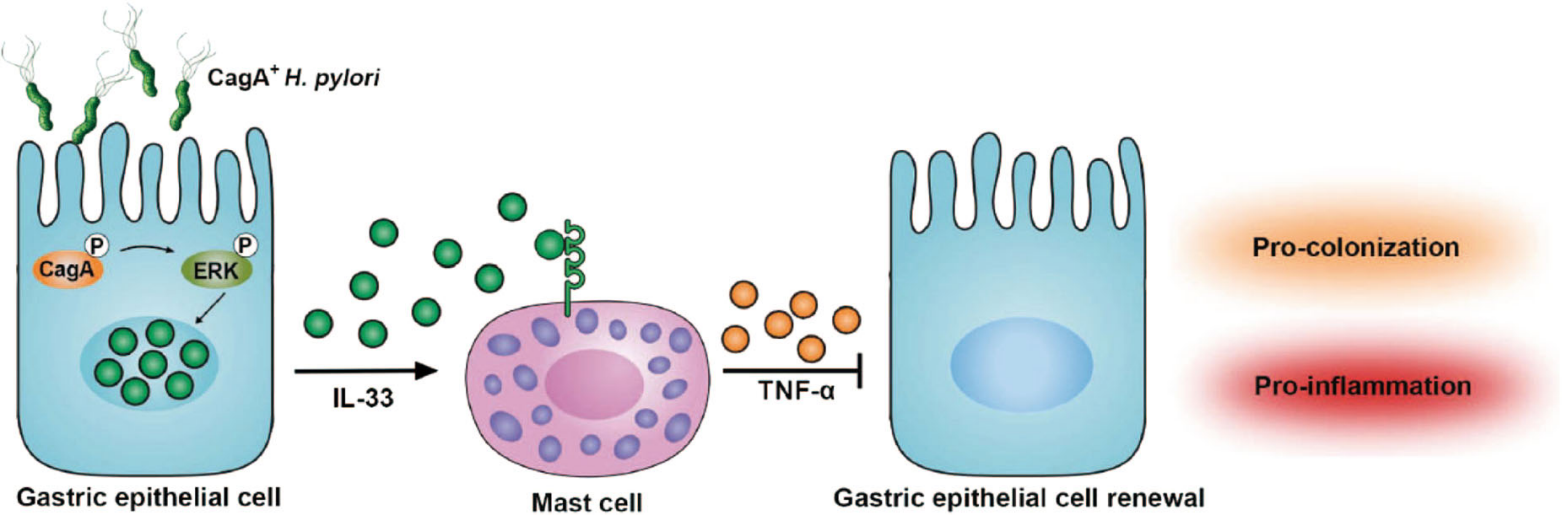
A proposed model of crosstalk among *H. pylori*, gastric epithelial cells, IL-33, mast cells, and TNF-α leading to inflammation and bacteria colonization in gastric mucosa during *H. pylori* infection. *H. pylori* stimulates gastric epithelial cells to secrete IL-33 via ERK pathway activation. Release of IL-33 from gastric epithelial cells induces ST2-expressing mast cells to produce TNF-α. Increased TNF-α disturbs the function of gastric epithelial cells by inhibiting gastric epithelial cell proliferation and promoting gastric epithelial cell apoptosis. This IL-33-TNF-α axis exerts a pathological effect, contributing to inflammation and bacteria colonization in gastric mucosa during *H. pylori* infection

In this study, we demonstrated a multistep model of inflammation during *H. pylori* infection within the gastric mucosa involving interactions between *H. pylori*, gastric epithelial cells, and mast cells via cytokines IL-33 and TNF-α (Fig. 7). In vivo and in vitro, we established that *H. pylori*-associated virulence factor *cagA* was essential to inducing IL-33 expression in gastric epithelial cells, which in turn promoted inflammation and *H. pylori* colonization. Moreover, the upregulated IL-33 induced mast cells to secrete TNF-α, which inhibited gastric epithelial cell renewal and aggravated gastritis. To our knowledge, this study is the first to consider the role of IL-33 and its association with mast cells in *H. pylori*-induced chronic inflammation and mast cell-derived TNF-α promoting gastritis in *H. pylori*-infected patients.

IL-33 was identified as a member of the IL-1 family^19^, which has various bioactive forms. Proteolytic processing played a crucial role in the regulation of IL-33 activity during inflammation, which could cleave full-length human IL-33 and generate mature forms^20^. For example, during infection, neutrophil proteases played important roles for IL-33 activation, but mast cell proteases predominated during allergic type-2 inflammation. In human, full-length IL-33 (IL-33_FL_) is a bioactive form that can induce ST2-dependent nuclear factor kappa-light-chain-enhancer of activated B cells (NF-κB) activity and cytokine production in target cells, however, multiple proteases are able to cleave IL-33_FL_ precursor to release shorter mature forms of 18–21 kDa, including IL-33_95–270_, IL-33_99–270_, and IL-33_109–270_. Interestingly, compared with IL-33_FL_, the biological activity of IL-33 mature forms increased significantly^20^. The immune cells that express membrane ST2 (ST2L), including tissue regulatory T cells (Tregs) and group 2 innate lymphoid cells (ILC2s), especially, mast cells, which are major targets of IL-33 and induce the production of different types of cytokines that play crucial roles in the exacerbation of allergic diseases and inflammation^17^. However, sequestration by soluble receptor sST2 is a crucial regulation of IL-33 activity and its activity can be limited by soluble receptor sST2^11^. IL-33 is considered to be associated with the pathophysiology of several allergic diseases by regulating mast cells. It has been reported that IL-33 directly promotes mast cell activation, which suggested that IL-33 presents important effect to impact mast cell airway smooth muscle crosstalk in asthma^21^. Besides, previous studies showed that IL-33 plays critical roles in acute infections. For instance, IL-33 was abundantly expressed in the skin of patients with *Staphylococcus aureus* infection^22^, and rapidly released to mobilize neutrophils to limit bacterial divisions^23^, and repair damaged tissue in acute polymicrobial sepsis^24^. However, a chronic infection state is more prominent in patients with *H. pylori*-associated gastritis and the potential role, if any, of IL-33 has not been elucidated. We decided to study the role of IL-33 in *H. pylori*-induced chronic infection. To extend our understanding of the interplay between IL-33 expression and *H. pylori* infection, using human gastric biopsy specimens infected with *H. pylori* and *H. pylori*-infected mouse models, we found that the expression of IL-33 was increased in patients and mice with *H*. *pylori* infection. Interestingly, our results showed that *H*. *pylori*-associated virulence factor *cagA* was necessary to induce maximal IL-33 expression by ERK signaling pathway. Our data were not entirely in keeping with the former studies^25^, in which the upregulation of IL-33 was independent of virulence factor *cagA*, although they found that the expression of IL-33 within *H. pylori*-infected patients was obviously higher compared with uninfected patients. One major difference (when compared with our current study) might be the different race studied or the insufficient subjects. Furthermore, some other studies showed increased IL-33 during early stage of *H. pylori* infection (Sydney strain 1) in the stomach^12^. But our results showed that IL-33 had a maximal expression in the mice gastritis model infected by *H*. *pylori* (11637 strain) at approximately the 2-month point p.i.

It is clear that a compound acute and chronic inflammatory response occurs simultaneously during *H. pylori*-associated gastritis, where multifarious immune cell types infiltrate gastritis tissues and these cells play complicated roles depending on the nature of the infection^26–28^. Recently, it has been reported that mast cells participate in *H. pylori*-associated gastritis^29,30^, however, the mechanism of infiltrated mast cell activation in *H. pylori*-associated gastritis remains unclearly. Earlier electron microscopy studies showed mast cell degranulation in *H. pylori*-infected gastric mucosa^31,32^, which were consistent with our earlier research^33^. However, the exact activation trigger was unclear. A recent study found that IL-33 could promote mast cell activation and release proinflammatory mediators in vitro^34,35^. Therefore, mast cells could potentially be activated directly by *H*. *pylori*, or indirectly by inflammatory factors during *H*. *pylori* infection. Our findings showed that the later mechanism is likely at play during chronic *H*. *pylori*-associated gastritis. More specifically, we showed that *H*. *pylori*-associated virulence factor *cagA* induced gastric epithelial cells to express IL-33, which in turn could induce mast cell activation and proinflammatory mediator TNF-α production. So, our results linked up *H*. *pylori* infection and mast cell activation for the first time.

It is known that TNF-α plays an important role in the pathogenesis of many chronic inflammatory diseases^36^. Besides the de novo synthesis TNF-α secretion showed here, mast cells are the only innate immune cells that can store and rapidly release preformed TNF-α^34,37^. In our study, mast cell-derived TNF-α could induce gastric epithelial cell apoptosis and suppress its renewal, whereas the effects could be blocked by TNF-α Ab. A previous report supported our results, which confirmed that mast cells were involved in chronic gastritis, meanwhile, the density of them was positively correlated with a high number of apoptotic epithelial cells^38^. And then, we also found the degree of inflammation and *H. pylori* colonization would be alleviated by TNF-α neutralizing Ab in vivo. Another research uncovered that *H. pylori* could increase the expression level of TNF-receptor 1, consequently, enhance the apoptosis of gastric epithelial cells mediated by this receptor^39^. The serum TNF-α level was also significantly elevated in *H.pylori*-induced gastritis in rats, which resulted in significant deterioration in stomach pathology, and added apoptotic epithelial cells^40^.

To sum up, our results suggest that mast cell may be a critical player in *H. pylori*-associated gastritis and IL-33 is also recognized as pivotal in inflammatory processes and is implicated in inflammatory diseases. Independent reports have shown that IL-33, mast cells, and TNF-α contribute to the inflammatory processes in *H. pylori*-induced gastritis^12,41,42^. Our findings provide insights into how IL-33, mast cells, TNF-α, and gastric epithelial cells may interact in *H. pylori*-induced gastritis. However, patients with *H. pylori*-induced gastritis have a diverse prognosis, with consequences ranging from an asymptomatic illness to fatal peptic ulcer^43^ or gastric cancer^44^. In consideration of the notable relationship between the expression level of IL-33 and the severity of gastric inflammation observed in *H. pylori*-infected patients (Fig. 4a), it is possible that IL-33 might serve as a novel diagnostic and prognostic biomarker for *H. pylori*-associated gastritis.

Although *H. pylori* can be effectively eradicated by oral antibiotics administration in most patients, it is worth noting that chronic gastritis generally persists even after successful *H. pylori* eradication therapy^45^. Therefore, it is beneficial to clinical prognosis, when treatments can manage the underlying inflammatory process. In view of this, our findings suggest several possible therapeutic targets, including IL-33, mast cells, and TNF-α. Our results are conductive to the understanding of the interactions among *H. pylori*, cytokines, and mast cells, but the conclusions are mostly based on experiments in vitro and in mice. Future clinical studies are necessary to investigate and verify the mechanisms in humans, which may lead to the application of novel pharmacologic approaches to resist this gastric pathogen.

## Materials and methods

### Patients and specimens

All the gastric biopsy specimens were gathered from 63 *H. pylori*-infected patients and 48 uninfected volunteers experienced upper esophagogastroduodenoscopy due to dyspeptic symptoms at XinQiao Hospital, Chongqing, China. *H. pylori* infection was first determined by rapid urease test of the biopsy specimens taken from antrum and by serology test for *H. pylori*-specific antibodies (Abs); the results were then conformed by real-time PCR for 16S ribosomal DNA (rDNA) as described below. For isolating human primary gastric mucosal tissue, fresh non-tumor gastric tissues (at least 5 cm distance from the tumor sites) were acquired from gastric tumor patients, and they had undergone surgical resection and determined as *H. pylori*-negative ones at the Southwest Hospital, Chongqing, China. For isolation of human primary gastric epithelial cells, fresh non-tumor gastric tissues were washed three times with Hank’s solution containing 1% fetal calf serum, and then, cut into pieces. All the specimen were gathered in RPMI-1640 medium containing DNase I (10 mg/ml) and collagenase IV (1 mg/ml), and then separated by using MACS Dissociator (Miltenyi Biotec). Then, the separated cell suspensions were incubated 0.5–1 h under continuous rotation at 37 °C. The single-cell suspensions were then filtered through a 70-μm cell strainer (BD Labware). Primary human gastric epithelial cells were sorted using Magnetic-activated cell sorting (MACS) columns and anti-CD326 magnetic beads (Miltenyi Biotec) from gastric tissue single-cell suspensions. The isolated human primary gastric mucosa tissue and human primary gastric epithelial cells were stimulated with *H. pylori* for further studies. None of the patients received radiotherapy or chemotherapy before sampling; patients with atrophic gastritis and multiple primary cancers, other infectious and autoimmune diseases, or received antibiotics treatments were excluded from this study. This research was authorized by the ethics committee of Southwest Hospital and XinQiao Hospital of Third Military Medical University, Chongqing, China. Each patient has signed the informed consent. Clinical characteristics of all the patients were described in Supplementary Table 1.

### Mice

The Animal Ethical and Experimental Committee of Third Military Medical University authorized all experiments and breeding with the specific approval and review. The specific pathogen free (SPF) female wild-type C57BL/6 (WT) mice were purchased from the Experimental Animal Centre of the Third Military Medical University. All mice were negative for pathogenic bacteria, parasites, and viruses. Mice were kept under SPF conditions with autoclaved water and food.

### Abs and other reagents

See Supplementary Table 2.

### Bacterial culture and mice infected with bacteria

*H. pylori* NCTC 26695, *H. pylori* NCTC 11637 (*cagA*-positive strain) (WT *H. pylori*-strain), and *cagA*-KO mutant *H. pylori* NCTC 11637 (*ΔcagA-*strain) were cultured in brain–heart infusion plates including rabbit blood (10%) with microaerophilic incubator at 37 °C. Bacteria were propagated in *Skirrow* broth containing 5% new born calf serum with gentle shaking with a microaerobic incubator for infecting mice. Centrifuging, collecting, and then adjusting bacteria to 10^9^ colony- forming units (CFU) per milliliter after a day’s culturing. Mice were inoculated orogastrically twice at 1-day interval with 3 × 10^8^ CFU bacteria after fast overnight. *Skirrow* broth was inoculated to the age-matched mice as mock controls. At each time point, five mice per group were used for the experiments.

### In vivo blockade of IL-33 or TNF-α

One hour before infection with WT *H. pylori-*strain, goat-anti-mouse IL-33 mAb (20 μg), or normal goat IgG (20 μg); rat-anti-mouse TNF-α mAb (20 μg) or rat IgG1 (20 μg) was afforded to each mouse via intraperitoneal injection. Before the mice were sacrificed at the indicated time, the administration was once a week. Finally, determining the *H. pylori* colonization and inflammation in the stomach.

### Evaluation of inflammation and bacterial load and isolation of single cells from tissues

The mice were sacrificed at the indicated time. The stomach was cut open from the greater curvature and half of the tissue was cut into four parts for RNA, DNA, tissue fixation for hematoxylin and eosin (H&E) staining and protein extraction, respectively. Two pathologists evaluated the degree of inflammation with a blinded manner, and then each section was received a score from 0 to 5 on the basis of the criteria^46^. DNA of the biopsy specimens were extracted with QIAamp DNA Mini Kit. As previously described^47^, *H. pylori* colonization was quantified by measuring *H. pylori*-specific 16S rDNA using specific primer and probe (Supplementary Table 3). Expression of 16S rDNA was measured using the TaqMan method. In the same specimen, the amount of mouse β2-microglobulin DNA was detected to normalize the data. According to a previous study^48^, the density of *H. pylori* was showed as the number of bacterial genomes per nanogram of host genomic DNA. Another half of stomach was used for isolation of single cells as described above. The isolated single cells were collected and analyzed by intracellular cytokine staining.

### Human gastric epithelial cell/tissue culture and stimulation

Human primary gastric tissues, gastric epithelial cells, or gastric epithelial cell line AGS cells were stimulated with WT *H. pylori*-strain and/or *ΔcagA-*strain, or *H. pylori* NCTC 26695 at different multiplicity of infection (MOI) or at different time points. In addition, for the signaling pathway inhibition experiments, AGS cells were pre-treated with 5 μl AG490 (a JAK inhibitor), BAY 11-7082 (an IκBα inhibitor), SB203580 (a mitogen-activated protein kinase (MAPK) inhibitor), SP600125 (a c-Jun N-terminal kinase (JNK) inhibitor), U0126 (MEK-1 and MEK-2 inhibitor), or Wortmannin (a PI3K inhibitor) (10 μM) for 2 h; then the cells were stimulated with WT *H. pylori-*strain (MOI = 100) for 24 h; harvested for RNA extraction with TRIzol reagent, and the expression of IL-33 was measured by real-time PCR.

### Mast cell cultures and treatments

The human mast cell line LAD2 (kindly provided by Professor Wei Zhang, Shenzhen Key Laboratory for Translational Medicine of Dermatology, China) were cultured in StemPro-34 medium supplemented with penicillin (100 U/ml)/streptomycin (100 µg/ml), human recombinant stem cell factor (hrSCF; 100 ng/ml), hrIL-6 (50 ng/ml) and l-glutamine (2 mM) as described previously^49^. Cells were cultured in a 37 °C 5% CO_2_ incubator. The femoral lavage of C57BL/6 mice were used to induce murine bone marrow-derived mast cells (BMMCs), which cultured in complete RPMI-1640 medium in the presence of mouse recombinant (mr) IL-3 (10 ng/ml), mrSCF (10 ng/ml), and l-glutamine (2 mM)^50^. The non-adherent cells were passaged every 3 days. Four weeks later, the mature mast cells were used for further experiments.

LAD2 cells, BMMCs, and CD8^+^ lymphocytes (sorting from PBMC by FACS) (1 × 10^6^ cells per milliliter) were stimulated with various concentrations of hrIL-33 or mrIL-33 (5–100 ng/ml) for 24 h. In other cases, LAD2 cells were stimulated with culture supernatants from cultured human primary gastric epithelial cells infected with WT *H. pylori-*strain. In some cases, blocking Ab for the IL-33 receptor ST2 (20 μg/ml) or a control IgG was added into LAD2 culture and incubated for 2 h before stimulation. Furthermore, IL-33 neutralizing Ab (20 μg/ml) or control IgG was added into the supernatants in some assays. After 24-h stimulation, the supernatants were harvested for enzyme-linked immunosorbent assay (ELISA).

### Gastric epithelial cell proliferation and apoptosis assays

The first primary gastric epithelial cell culture supernatants derived from WT *H. pylori-*strain-stimulated human primary gastric epithelial cells were collected to stimulate LAD2 cells for 24 h. Then, the second mast cell culture supernatants were again collected as treatment liquids. Human gastric epithelial cell line AGS was stimulated with the second mast cell culture supernatants in the presence of a human TNF-α neutralizing Ab (20 μg/ml) or control IgG1 (20 μg/ml). In other cases, AGS cells were stimulated with rhTNF-α (100 ng/ml) in the culture system. Cell proliferation was measured by CCK-8 and cell apoptosis was detected by APO-Direct Apoptosis Detection or Annexin V Apoptosis Detection Kit I for 72 h in accordance with the manufacturer’s instructions.

### Real-time PCR

Extracted RNA from biopsy specimens and cultured cells were reverse-transcribed to complementary DNA by PrimeScriptTM RT reagent Kit. Real-time PCR was measured with the Real-time PCR Master Mix in accordance with the manufacturer’s specifications on the IQ5 (Bio-Rad). Expression of IL-33 and TNF-α was detected using the SYBR green method with the respective primers (Supplementary Table 3). For mouse samples, mouse β2-microglobulin served as the normalizer, and uninfected stomach served as a calibrator. For human samples, uninfected control cells served as a calibrator, and human glyceraldehyde 3-phosphate dehydrogenase (GAPDH) served as the normalizer. The relative gene expression was showed as fold change calculated by the ΔΔCt method.

### Western blots

Equivalent amounts of cell or tissue lysates were resolved in 10% sodium dodecyl sulfate–polyacrylamide gel electrophoresis gels, proteins were then transferred onto polyvinylidene difluoride membranes and western blots were performed. Human or mouse IL-33 was detected with rabbit anti-IL-33 Abs; human p-ERK1/2, ERK1/2, and GAPDH were detected, respectively, with rabbit anti-p-ERK1/2 Abs, rabbit anti-ERK1/2 Abs, and mouse anti-GAPDH Abs, then followed by incubation with horseradish peroxidase-conjugated secondary Abs. Detected proteins were shown with a SuperSignal® Extended Duration Substrate kit.

### Enzyme-linked immunosorbent assay

Human and mouse gastric tissues from specimens were collected, homogenized in 1 ml sterile Protein Extraction Reagent, centrifuged, and harvested. Tissue supernatants were assayed using the IL-33 ELISA Kit. Human gastric biopsy specimens of *H. pylori*-infected patients and uninfected volunteers cultured in vitro for 24 h, and then the supernatants centrifuged and harvested for detecting sST2 by ELISA Kit. Supernatants from mast cells and CD8^+^ lymphocytes were assayed using the TNF-α ELISA Kit according to the manufacturer’s instructions.

### Immunohistochemistry

Human gastritis tissue samples were fixed with paraformaldehyde and embed with paraffin, and then were cut into 5 µm sections. For immunohistochemical staining, mouse anti-human tryptase Ab was used as the primary Ab. And then sections were stained by EnVision G2 System/AP Rabbit/Mouse. After that, all the sections were counterstained with hematoxylin and reviewed using a microscope (Nikon Eclipse 80i; Nikon).

### Immunofluorescence

Paraformaldehyde-fixed sections of *H. pylori*-infected human and mouse tissues were washed in phosphate-buffered saline and blocked with 20% goat serum for 30 min, and stained for IL-33, CD326, CD8, ST2, tryptase, and TNF-α. Slides were examined with a confocal fluorescence microscope (LSM 510 META, Zeiss).

### Flow cytometry

Cells were stained with Abs for surface markers or with isotype control Abs. Intracellular cytokine staining was performed after fixation and permeabilization using Perm/Wash solution. The cells were analyzed by multicolor flow cytometry with a FACSCanto II (BD Biosciences). Data were analyzed with FACSDiva software (BD Biosciences) or FlowJo software (TreeStar).

### Statistical analysis

All statistical analysis was used by SPSS statistical software (V.13.0). Results are shown as the mean value ± SEM. Generally, Student's *t*-test or the Mann–Whitney *U*-test was used to analyze the differences between two groups, depends on the variances differed or not. Correlations analyses were done using Pearson correlation analysis and linear regression analysis. All data were analyzed using two-tailed tests, and differences were considered to be statistically significant when *P* < 0.05.

## Electronic supplementary material

Supplementary Figure Legends

Supplementary Table 1

Supplementary Table 2

Supplementary Table 3

Supplementary Figure 1

Supplementary Figure 2

Supplementary Figure 3
