# Supplementary material for: Helicobacter pylori-induced IL-33 modulates mast cell responses, benefits bacterial growth, and contributes to gastritis
Source: Cell Death Dis. 2018 Apr 25;9(5):457. doi: 10.1038/s41419-018-0493-1 (PMC5915443; doi:10.1038/s41419-018-0493-1)
Supplement: Supplementary file 1 — Supplementary Figure Legends [file 41419_2018_493_MOESM1_ESM.doc]

Supplementary Figure 1. *H. pylori* infected-gastric epithelial cells produce IL-33. (**a**) IL-33 mRNA expression in *H. pylori* 26695-infected and uninfected AGS cells (MOI=100) was compared (n=3). (**b**) IL-33 mRNA expression in WT *H. pylori*-infected, *ΔcagA*-infected, and uninfected AGS cells (MOI=100, 24 h) was compared (n=3). (**c**) IL-33 mRNA expression in WT *H. pylori*-infected, *ΔcagA*-infected, and uninfected primary gastric epithelial cells (MOI=100, 24 h) was compared (n=3). (**d**) Production of sST2 protein in gastric mucosa of *H. pylori*-infected patients (n=8) and uninfected donors (n=8) was analyzed by ELISA. ***P*<0.01, n.s. p>0.05 for groups connected by horizontal lines compared.

Supplementary Figure 2. IL-33 promotes TNF-α production from mast cells during *H. pylori* infection. BMMCs cells were and stimulated **with** IL-33. Production of TNF-α protein (**a**) and expression of TNF-α mRNA (**b**) were analyzed by ELISA (n=3) or compared (n=3). **P*<0.05, ***P*<0.01 for groups compared with unstimulated cells. (**c**) Representative immunofluorescence images showing CD8^+^ST2^+^ cell infiltration in gastric mucosa of *H. pylori*-infected patients. Green, CD8; red, ST2; and blue, DAPI-stained nuclei. Scale bars: 50 microns. (**d**) LAD2 cells and CD8^+^ lymphocytes were stimulated **with medium and** IL-33 (10 ng/ml). Production of TNF-α protein was analyzed by ELISA (n=3). **P*<0.05, ***P*<0.01 for groups compared with unstimulated cells.

Supplementary Figure 3. TNF-α promotes inflammation and bacteria colonization in gastric mucosa during *H. pylori* infection. (**a**) AGS cells were stimulated with TNF-α, as described in Methods. The proliferation of AGS cells were analyzed (n=3). (**b**) Dynamic changes of TNF-α mRNA expression in WT *H. pylori*-infected, *ΔcagA*-infected, and uninfected mice. (**c** and **d**) AGS cells were stimulated with TNF-α, as described in Methods. The apoptosis of AGS cells were analyzed by annexin V (**c**) and deoxyuridine triphosphate nucleotides (dUTP) (**d**) detection (n=3). **P*<0.05, ***P*<0.01 for groups connected by horizontal lines compared, or compared with uninfected mice.
