# Supplementary material for: Helicobacter pylori-induced IL-33 modulates mast cell responses, benefits bacterial growth, and contributes to gastritis
Source: Cell Death Dis. 2018 Apr 25;9(5):457. doi: 10.1038/s41419-018-0493-1 (PMC5915443; doi:10.1038/s41419-018-0493-1)
Supplement: Supplementary file 2 — Supplementary Table 1 [file 41419_2018_493_MOESM2_ESM.doc]

**Supplementary Table 1** Clinical characteristics of patients

| Variables | *H. pylori-*infected | Uninfected |
| --- | --- | --- |
| Age (median, range)  Sex (male/female) | (45 year, 24-69 years)  30/33 | (49 year, 18-65 years)  20/28 |

Exclusion criteria were: previous treatment for H. pylori infection, use antibiotics and/or acid secretion inhibitors during the 2 months before the study, use of anticoagulant drugs in the last week, long-term use of corticosteroids or anti-inflammatory drugs, severe concomitant cardiovascular, respiratory or endocrine diseases, gastrointestinal malignancy, clinically significant renal or hepatic disease, haematological disorders, previous gastro-oesophageal surgery, pregnancy or lactation, history of allergy to any of the drug used in the study, alcohol abuse, drug addiction, and severe neurological or psychiatric disorders.
