# Supplementary material for: Helicobacter pylori-induced IL-33 modulates mast cell responses, benefits bacterial growth, and contributes to gastritis
Source: Cell Death Dis. 2018 Apr 25;9(5):457. doi: 10.1038/s41419-018-0493-1 (PMC5915443; doi:10.1038/s41419-018-0493-1)
Supplement: Supplementary file 3 — Supplementary Table 2 [file 41419_2018_493_MOESM3_ESM.doc]

**Supplementary Table 2** Antibodies and other reagents

| Antibodies and reagents | Manufacturers |
| --- | --- |
| | Antibodies for flow cytometry  anti-CD326-FITC  anti-IL-33-PE  anti-CD8-PE  Antibodies for immunohistochemical staining  anti-human tryptase  EnVision™ G2 System/AP Rabbit/Mouse (Permanent Red)  Antibodies for immunofluorescence  goat anti-human IL-33  goat anti-human ST2/IL-33R  mouse anti-human tryptase  rabbit anti-human TNF-a  rabbit anti-mouse IL-33  rabbit anti-mouse EpCam  rabbit anti-human CD8  rabbit anti-goat-TRITC  goat anti-mouse-FITC  goat anti-rabbit-TRITC  goat anti-rabbit-FITC  DAPI Staining Solution  Antibodies for neutralizing and blocking  Anti-human TNF-α  Mouse IgG1, κ isotype ctrl  Anti-mouse IL-33 Normal goat IgG control Anti-mouse TNF-α  Rat IgG1, κ Isotype Ctrl  Antibodies for western blot  anti-human IL-33  anti-human p-Erk1/2  anti-human Erk  ELISA kits  Human IL-33  Mouse IL-33  Human TNF-α  Mouse TNF-α  Human sST2  Reagents for signaling pathways inhibition  MEK-1 and MEK-2 inhibitor U0126  JAK signaling inhibitor AG490  IκBα inhibitor BAY 11-7082  JNK inhibitor SP600125  MAPK inhibitor SB203580  PI3K inhibitor Wortmannin  CCK-8 Kits  PE Annexin V Apoptosis Detection Kit I  APO-Direct Apoptosis Detecion | Biolegend | | --- | --- | | Collagenase IV |  | | DNase I  DMSO  Protein Extraction Reagent  SuperSignal® West Dura Extended Duration Substrate kit  Fetal calf serum (FCS)  Penicillin/Streptomycin  RPMI-1640  F12  StemPro®-34 SFM  L-glutamine  Trypsin 0.25%(1X) solution  Trizol  PrimeScriptTM RT reagent Kit  Real-time PCR Master Mix  QIAamp DNA Mini Kit  Premix Ex Taq*™*(Probe qPCR)  All recombinant human/mouse cytokines |  | | |  | | --- | | Biolegend | | R&D Systems  Biolegend  Abcam  Dako  R&D Systems  R&D Systems  Abcam  Abcam  Abcam  Abcam  Abcam  Zhongshan Biotechnology  Zhongshan Biotechnology  Zhongshan Biotechnology  Zhongshan Biotechnology  Beyotime  Biolegend  Biolegend  R&D Systems  R&D Systems  Biolegend  Biolegend  Abcam  Cell signaling technology  Cell signaling technology  Biolegend  Biolegend  Biolegend  Biolegend  Raybiotech  Merk Millipore  Merk Millipore  Merk Millipore  Merk Millipore  Merk Millipore  Merk Millipore  Dojindo  BD Biosciences  Invitrogen  Gibco  Sigma-Aldrich  Sigma-Aldrich  Pierce  Thermo  Gibco  Gibco  Hyclone  Hyclone  Thermo  Gibco  Hyclone  TaKaRa  TaKaRa  Toyobo  QIAGEN  TaKaRa  PeproTech | |

FITC, Fluorescein isothiocyanate; PE, phycoerythrin; TRITC, Tetramethylrhodamine; IL, interleukin; EpCam, epithelial cell adhesion molecule.
