# Supplementary material for: Helicobacter pylori-induced IL-33 modulates mast cell responses, benefits bacterial growth, and contributes to gastritis
Source: Cell Death Dis. 2018 Apr 25;9(5):457. doi: 10.1038/s41419-018-0493-1 (PMC5915443; doi:10.1038/s41419-018-0493-1)
Supplement: Supplementary file 4 — Supplementary Table 3 [file 41419_2018_493_MOESM4_ESM.doc]

**Supplementary Table 3** Primer and probe sequences for real-time PCR analysis

| Gene | Primer of probe | Sequence 5′→3′ |
| --- | --- | --- |
| *H. pylori* 16S rDNA  Mouse β2-microglobulin  Human IL-33  Human TNF-α  Human GAPDH  Mouse IL-33  Mouse TNF-α  Mouse β-actin | forward  reverse  probe  forward  reverse  probe  forward  reverse  forward  reverse  forward  reverse  forward  reverse  forward  reverse  forward  reverse | TTTGTTAGAGAAGATAATGACGGTATCTAAC  CATAGGATTTCACACCTGACTGACTATC  CGTGCCAGCAGCCGCGGT  CCTGCAGAGTTAAGCATGCCAG  TGCTTGATCACATGTCTCGATCC  TGGCCGAGCCCAAGACCGTCTAC  GCCAACAACAAGGAACACTCTG  CACTCCAGGATCAGTCTTGCAT  CAATGGCGTGGAGCTGAGAG  GTCTGGTAGGAGACGGCGAT  ACCCAGAAGACTGTGGATGG  CAGTGAGCTTCCCGTTCAG  CGCTACTATGAGTCTCCCTGTCCT  GAAGAAGGCCTGTTCCGGAGG  AACCTCCTCTCTGCCGTCAA  AAGTAGACCTGCCCGGACTC  AGTGTGACGTTGACATCCGT  GCAGCTCAGTAACAGTCCGC |

For the probes, a FAM fluorescent reporter is coupled to the 5' end, and a TAMRA quencher is coupled to the 3'
end.
