# Supplementary figures and images for: Helicobacter pylori-induced IL-33 modulates mast cell responses, benefits bacterial growth, and contributes to gastritis
Source: Cell Death Dis. 2018 Apr 25;9(5):457. doi: 10.1038/s41419-018-0493-1 (PMC5915443; doi:10.1038/s41419-018-0493-1)

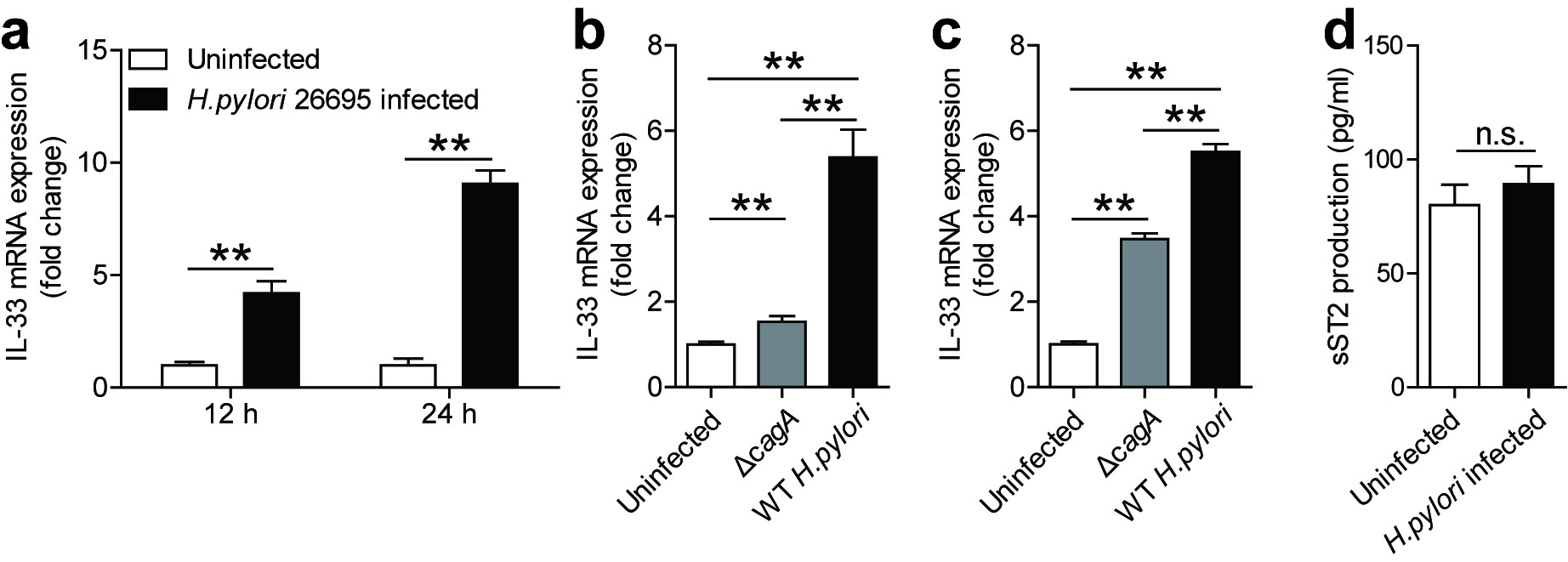

Supplement: Supplementary file 5 — Supplementary Figure 1 [file 41419_2018_493_MOESM5_ESM.jpg]

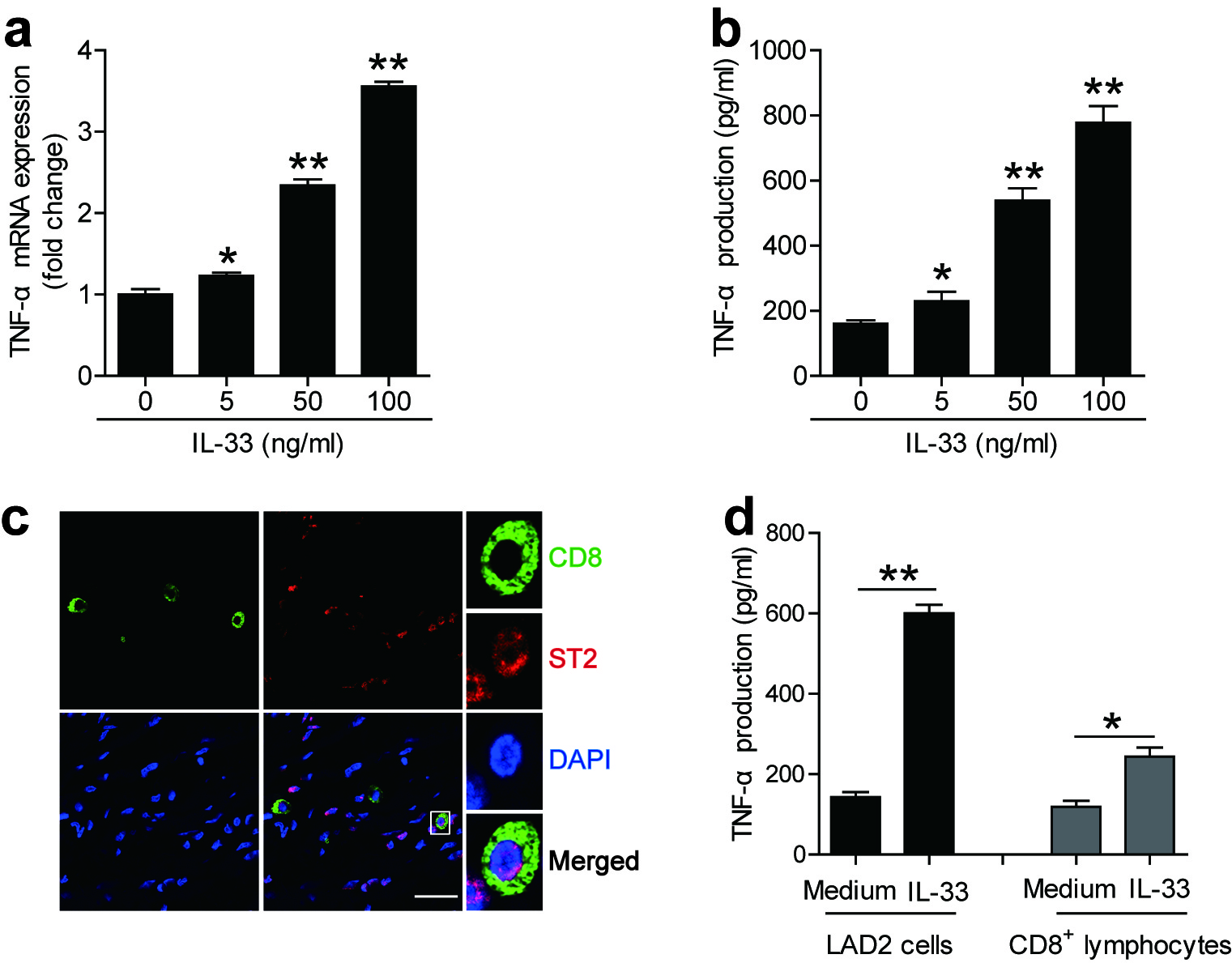

Supplement: Supplementary file 6 — Supplementary Figure 2 [file 41419_2018_493_MOESM6_ESM.jpg]

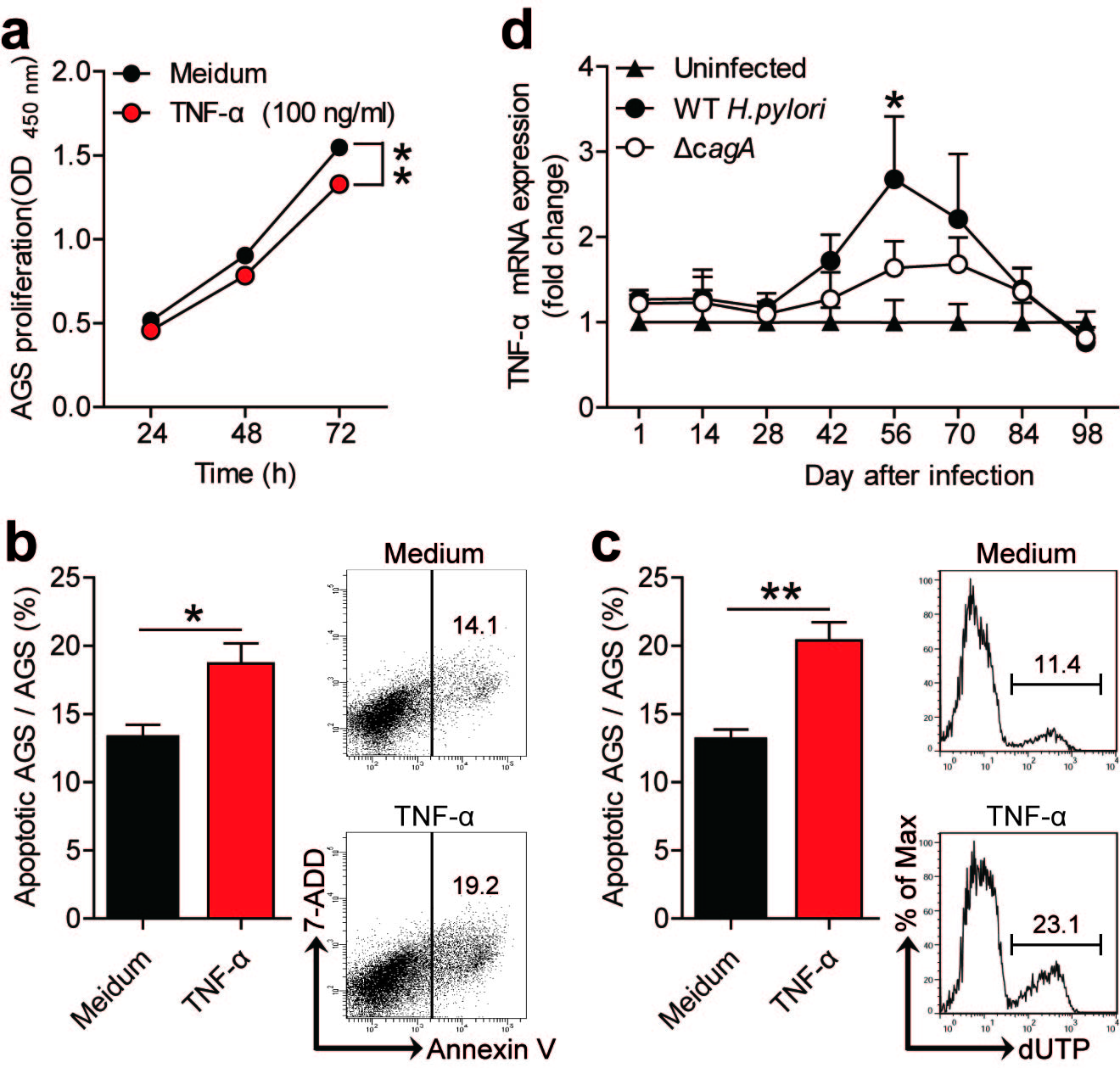

Supplement: Supplementary file 7 — Supplementary Figure 3 [file 41419_2018_493_MOESM7_ESM.jpg]
